# Stable isotopic profile of commercial tank milk in relation to grassland based feed proportions in dairy herd diets

**DOI:** 10.1038/s41598-025-97041-z

**Published:** 2025-04-12

**Authors:** Amy Birkinshaw, Michael Sutter, Rudi Schäufele, Michael Kreuzer, Beat Reidy

**Affiliations:** 1 School for Agricultural, Forest and Food Sciences (HAFL), Bern University of Applied Sciences (BFH), Laenggasse 85, Zollikofen, 3052 Switzerland; 2 TUM School of Life Sciences, Crop Physiology, Alte Akademie 12, 85354 Freising, Germany; 3 Institute of Agricultural Sciences, ETH Zurich, Eschikon 27, Lindau, 8315 Switzerland

**Keywords:** Cattle, Herbage, Carbon, Oxygen, Nitrogen, Hydrogen, Plant sciences, Environmental sciences

## Abstract

Ours is the first proof-of-concept study to attempt to define stable isotopic ratio (δ) thresholds to ascertain the dietary proportion of grassland-based feeds (GBF) in commercial tank milk. Additionally, the isotopic profile of commercial tank milk produced in Switzerland was characterized. We collected 217 tank milk samples, from 21 dairy farms, over one calendar year to reflect and incorporate seasonal variations in feeding strategies. Thus, a wide range of feeding strategies based on contrasting proportions of total GBF (28–99%) and grazed herbage (0–96%) were represented. Based on mixed-model multiple regression, there was a significant association of δ^13^C and δ^18^O values of tank milk and the dietary proportions of GBF and grazed herbage, respectively. For every 10% increase in total GBF, δ^13^C decreased by 0.673 (R^2^ = 0.57) and for every 10% increase in grazed herbage, δ^18^O increased by 0.125 (R^2^ = 0.51). With this information, δ^13^C and δ^18^O values of bulk milk may be suitable starting points for the development of reliable indicators to assess and control the proportion of total GBF and grazed herbage in dairy farm diets. Commercial tank milk produced in Switzerland has a mean value of − 26.0, 5.69, − 7.03 and − 53.4 for δ^13^C, δ^15^N, δ^18^O and δ^2^H, respectively.

**Supplementary Information:**

The online version contains supplementary material available at 10.1038/s41598-025-97041-z.

## Introduction

As the global population continues to increase, sustainable food and production systems are an absolute necessity^1^. In order to feed a population of 10 billion by 2050, it is essential to master the trade-offs between food security and sustainability^2^. Bovine milk is considered a complete protein as it contains all essential amino acids and is one of the top ranked foods in terms of nutrient-to-calorie ratio for numerous amino acids, calcium, phosphorus and riboflavin^3^. Currently, world milk production (predominantly bovine) is forecasted to increase by 1.7% per annum to 1’020 million metric tons by 2030^4^.

Dairy production from grassland-based systems, where dairy herds are predominantly maintained on fresh grass, grass silage, lucerne and grass hay, is a sustainable feeding strategy in temperate countries with abundant natural and semi-natural grasslands. In these systems, non-arable or marginal land not fit for human food production can be used as forage for dairy cows^5^. Dairy cows have the unique ability to transform human-inedible fiber into high quality milk proteins^6^ without competing for human-edible crops such as cereals and soybean^7^. Consequently, grassland-based dairy production may have lower environmental impacts in terms of resource inputs and pollutant output than conventional systems^8^ and despite increasing livestock numbers and management intensification grasslands have proven climate neutral^9^. Consumers too play a crucial role in reducing the environmental burden of their food choices^10^ and they are becoming increasingly aware of the sustainability, socio-economic, environmental and animal welfare implications their dietary preferences pose^11^. Dairy produced under “grass-fed” labels fetches premium prices as consumers perceive it to be more natural and more sustainable compared to conventional dairy production^12^. However, to date there is no global definition for “grass-fed milk” with countries and labels imposing their own “grass-fed” standards. These often include indirect measurements such as the amount of time or number of days spent on pasture or the total pasture area available divided by the herd. Even the scientific literature regarding the authentication of “grass-based” feeding envelops a wide range of scenarios including primarily grass-based versus maize- or concentrate-based diets, grazed grass versus cut-and-carry fresh herbage indoor feeding and fresh versus ensiled grassland-based feeds^13^. Such diversity and lack of clarity has led to an international call for a grassland-based feeding standard that can be directly and easily measured^14,15^.

Stable isotope ratio analysis is of particular interest as an authentication tool in this context as it has the combined ability to provide detailed information about dietary intake as well as the geographical origin of dairy herds from which milk and dairy products are derived^16^. Stable isotopes in nature fractionate in predictable and reproducible manners from the diet (carbon (C) and nitrogen (N)), environment and geographical location (oxygen (O) and hydrogen (H)) which are then reflected in the milk^11^. Stable isotope ratio analysis has been successfully applied to differentiate the main constituents of dairy herd diets in terms of C_3_ (temperate grasses) versus C_4_ (maize) plants^17,18^, levels of cereal concentrate input^16^ and the proportion of maize in the diet^19^.

Stable isotope ratio analysis has also been suggested as a key technique to evaluate milk production systems “grass-fed” claims^11,17^. However, to the best of our knowledge no other study has defined stable isotopic ratio thresholds in a first-step, proof-of-concept study to ascertain the proportion of grassland-based feeds (GBF) in a dairy herd’s diet. Additionally, the isotopic profile of milk produced in Switzerland has not yet been reported which due to the unique geographic characteristics should provide a rare profile. Therefore, with a possible future grassland-based “sustainability indicator” in mind, we aimed to (1) define any significant linear relationships between δ^13^C, δ^15^N, δ^18^O and δ^2^H and intake of GBF, (2) assess the suitability of these four stable isotope ratios as possible indicators for the GBF proportion in the diets of dairy herds and (3) concomitantly characterize the isotopic profile of milk produced in Switzerland. We hypothesized that decreases in δ^13^C values and increases in δ^18^O values, with increasing proportions of GBF and fresh herbage in the diets of dairy herds, respectively, may be reliable determinants of diet composition. This information may be useful in attempting to guarantee a certain proportion of grass-based feeds and/or fresh herbage in the rations of dairy cows for policy makers, milk producers, milk buyers and processors, farm assurance schemes and dairy consumers alike.

## Materials and methods

### Farm and feed data

**Fig. 1:**
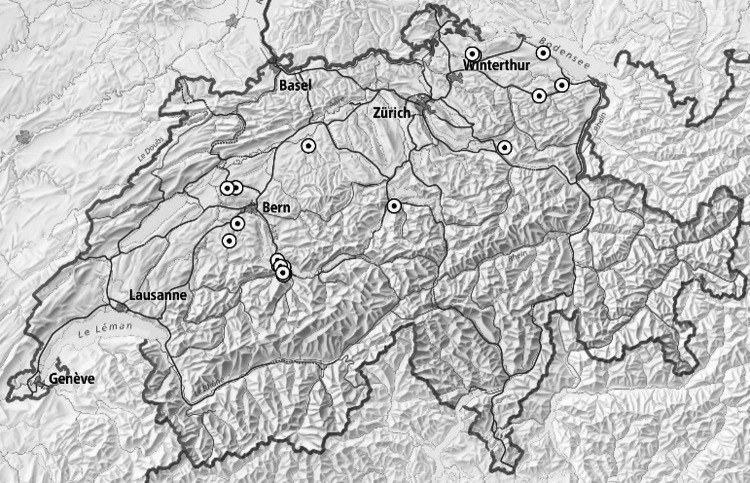
Location of the 21 commercial dairy farms included in the study. (Created with QGIS, version 3.14 Pi, https://qgis.org/download/).

The present study, a sub-set of the data used in Birkinshaw et al.^20^, was conducted in Switzerland from January to December 2020 to reflect and incorporate seasonal variations in farm feeding strategies. Twenty-one commercial dairy farms were selected to represent a wide range of feeding strategies based on contrasting dietary proportions of GBF (28–99%, annual mean = 72%), grazed herbage (0–96%, annual mean = 21%) and whole crop maize (0–52%, annual mean = 15%) from diverse locations within Switzerland (Fig. 1). Informed consent was obtained by signature for each farm before inclusion in the study. The realized proportions of individual feeds on the farms were determined and verified using a series of cross-checks described in detail by Birkinshaw et al.^20^. A complete overview of the main feed components fed to animals on each of the 21 farms included in the present study is given in Supplementary Table S1. This table also includes concentrates and other commercial feed products, which contributed an average of ≤ 10% to the diet and were considered to have a negligible effect on the stable isotope ratios of commercial tank milk.

### Commercial milk sampling and stable isotope analysis (C, N, O, H)

Twice monthly, routine chilled (4 °C) commercial tank milk samples (50 ml) were collected from each farm after morning milking and thorough mixing of the milk in the milk tank. All samples contained morning and the previous evening’s milk. Within 8 h, samples were transported chilled and frozen at ‒20 °C until stable isotope ratio analysis.

For analysis, the two monthly samples were pooled in equal proportions to form one sample per farm, per month. Measurements of the ratios of ^13^C/^12^C (δ^13^C) and ^15^N/^14^N (δ^15^N) were performed on whole, thawed, raw milk. Samples were homogenized by vortexing for 1 min (Vortex Genie 2, Scientific Industries Inc., Bohemia, NY, USA) before pipetting a 5 µl aliquot into ultra-clean tin cups (4 × 6 mm). Subsequently, samples were dried for 24 h at 60 °C and then combusted with an elemental analyzer (EA1100, Carlo Erba Instrumentatione, Milan, Italy). The oxidation tube was packed with CrO_3_, silver-coated CoO and quartz wool; and the reduction tube with CuO, elemental Cu and quartz wool (all chemicals: IVA Analysetechnik, Meerbusch, Germany). The elemental analyzer was interfaced (Conflo III, Finnigan MAT, Bremen, Germany) to an isotope ratio mass spectrometer (Delta Plus, Finnigan MAT, Bremen, Germany) operating in continuous flow mode. Samples were measured against a working standard CO_2_ reference gas (purity = 4.5; Westfalen AG, Münster, Germany) calibrated against the secondary isotope standards, IAEA-CH6 and IAEA-CH3 (sucrose and cellulose, respectively; both provided by the International Atomic Energy Agency (IAEA), Vienna, Austria). A solid internal laboratory standard (a finely ground protein powder) was run after every tenth sample as a control and to account for possible drift during the measurement runs. Long term precision (referring to the reproducibility of replicate sample measurements) of this internal standard for the measurement period was 0.11‰ for δ^13^C and 0.15‰ for δ^15^N.

Measurements of the ratios of ^18^O/^16^O (δ^18^O) and ^2^H/^1^H (δ^2^H) were conducted by the on-line, continuous flow method described in detail by Sharp et al.^21^. Milk water was extracted from each raw milk sample with a custom-built cryogenic vacuum distillation apparatus. Once extracted, 300 µl aliquots were analyzed using a cavity ring-down spectroscopy analyzer coupled to an A0211 high precision vaporizer (Piccaro Inc., Sunnyvale, Ca, USA) set at 110 °C. Five to 12 replicate injections (1 µl each) were conducted per sample and an average of the last two measurements was used. Post-processing correction was applied by applying the ChemCorrect™ software (v.12.0, Piccaro Inc.) to eliminate the influence of possible dissolved organic contaminants^22^. Two internal laboratory water standards were run after every 20 samples to correct for drift. These standards were previously calibrated against the Vienna-Standard Mean Ocean Water, Vienna-Greenland Ice Sheath Precipitation and Vienna-Standard Light Antarctic Precipitation standards provided by the IAEA using the same procedure as applied in sample analysis. Long term precision (referring to the reproducibility of replicate sample measurements) of the internal laboratory water standards for the measurement period were 0.12‰ (0.012%) for δ^18^O and 0.67‰ (0.067%) for δ^2^H.

The results of the stable isotope measurements are expressed in per mil (‰) against international standards as:

δ = $$\:(\frac{R\:sample}{\:R\:standard\:}-1)$$ × 1000

where R = ratio of heavy to light isotope of the element and the standards (Vienna Pee Dee Belemnite for δ^13^C, Vienna Air for δ^15^N and Vienna-Standard Mean Ocean Water for δ^18^O and δ^2^H).

### Statistical analyses

All statistical analyses were performed using R (v.4.3.0) and the lmerTest^23^, multcomp^24^ and report^25^ packages.

To establish significant linear relationships between dietary components and stable isotope ratios and assess the suitability of stable isotope ratios as possible indicators for GBF proportions, a linear mixed effects model was applied. The best linear mixed effects model for each stable isotope ratio was selected using the lowest Akaike Information Criterion from a set of five models with increasing complexity. These models were generated by the sequential addition of four explanatory variables (Latitude, Longitude, Month, and the interaction between Month and Diet) to the existing dietary component model. Altitude was not included in our models as the weak correlations between isotopic values (δ¹³C positively, δ¹⁵N negatively) and elevation were not strong enough to meaningfully improve model performance. Latitude and longitude were included as several European climates create distinct climatic gradients across Switzerland. Mild, moist air masses (influenced by the North Atlantic Drift) come from the west, while dry, cold air arises from Northern Arctic and Eastern continental areas, with dry, cold conditions in winter and warm in summer. Moist and relatively warm air from the Mediterranean flows northwards. These air masses interact with the complex Swiss topography, generating a longitudinal spatial pattern in temperature and precipitation that influence ecological processes and isotopic variation even in a small country. Month was included instead of season to capture finer temporal variation, as it offers more detailed resolution compared to season as a broader classification. The most complex model reads as:

Y_ij_ = b_0_ + b_1_×Diet_Cij_ + b_2_×Latitude_i_ + b_3_×Longitude_i_+ b_4_×Month_j_ + b_5_×Month_j_×Diet_Cij_ + u_i_ + e_ij_.

where Y_ij_ = the stable isotope ratio (δ^13^C, δ^15^N, δ^18^O, δ^2^H); i = farm ID, with a random effect and j = individual milk samples within each farm; b_0_ = overall mean; b_1_,_2_,…_5_ = regression coefficients of the observed effects of the dietary component (Diet_Cij,_ total GBF or grazed herbage or maize), farm location (Latitude_i_ and Longitude_i_), month of the year (as a categorical variable), the interaction between the month (January to December) of the year and the dietary component (Month_j_×Diet_Cij_). u_i_ = the random intercept for farm and e_ij_ is the random error of the model. Including latitude and longitude ensured that the effects of dietary proportions were corrected for geographic differences between farms. The model’s explanatory power is reported using marginal R^2^ (variance explained by fixed effects) and conditional R^2^ (variance explained by both fixed and random effects).

Additionally, a one-way analysis of variance with post-hoc Tukey HSD tests was performed to (i) determine the effect of season on each of the stable isotope ratios (δ^13^C, δ^15^N, δ^18^O, δ^2^H) of commercial tank milk and (ii) assess the impact of defined grassland-based feeding strategies on milk δ^13^C values. For this purpose, each farm was allocated to one of four defined feeding strategies based on the mean proportion of total GBF (1) ≤ 60%, 2) 61–74%, 3) 75–84% and 4) 85–100%) in the diet.

Descriptive statistics (mean, standard deviation and range) were used to characterize the isotopic profile of milk produced in Switzerland.

## Results

Based on the results of the present study, commercial tank milk in Switzerland has a mean (± standard deviation) of ‒26.0‰ (± 2.4), 5.69‰ (± 0.86), ‒7.03‰ (± 1.72) and ‒53.4‰ (± 9.0) for δ^13^C, δ^15^N, δ^18^O and δ^2^H, respectively. A detailed profile of the stable isotope ratios of commercial tank milk is provided for each of the 21 farms in Supplementary Table S2. Table 1 presents regression coefficients from the mixed models for the three dietary components (total grassland-based feeds, grazed herbage and maize) across all four stable isotope ratios; whereas, Table 2 is a simple descriptive summary showing the mean and range of the four isotope ratios, along with the P value from a one-way ANOVA, where season (not month) is the explanatory variable.

**Table 1:** Regression coefficients for commercial tank milk stable isotope ratios (C, N, O, H) in relation to the proportions of grassland-based feed and total maize in the diet. GBF, grassland-based feed.

Milk stable isotope ratio	Intercept	Grassland-based feeds		Maize	Other fixed effects				Model		
	b_0_	Total	Grazed	Total	Month	Month×GBF	Latitude	Longitude	Marginal R^2^	Conditional R^2^	*P* value
δ^13^C	40.6	– 6.73^***^			– ^1^	–	−1.31	–	0.305	0.574	0.096
	–83.80		0.48		–1.86^***^	–	1.47	–1.31	0.101	0.679	0.044
	–27.4			9.71^***^	–	–	–	–	0.361	0.594	< 0.001
δ^15^N	6.19	–0.459			0.481^**April^	–	–0.124	0.693^*^	0.299	0.737	0.002
	1.53		0.352^†^		0.332^**April^	–	–0.028	0.676^*^	0.338	0.697	0.002
	8.14			0.838	0.479^**April^	–	–0.184	0.747^*^	0.299	0.745	0.001
δ^18^O	–63.3	0.646			2.13^***^	–	1.27^*^	–0.651^*^	0.450	0.489	< 0.001
	–57.5		1.25^**^		1.79^***^	–	1.13^†^	–0.526^†^	0.474	0.507	< 0.001
	–69.9			3.34^†^	3.04^***^	–9.66^***July^	1.41^*^	–0.66^*^	0.511	0.553	< 0.001
δ^2^H	–457	4.09			9.04^***^	–	9.28^*^	–5.27^**^	0.482	0.531	< 0.001
	–421		6.24^**^		7.42^***^	–	8.46^*^	–4.58^**^	0.497	0.555	< 0.001
	–433			–1.91	9.42^***^	–	8.81^*^	–5.16^**^	0.477	0.528	< 0.001

^***^P < 0.001, ^**^P < 0.01, ^*^P < 0.05, ^†^*P* < 0.10. Month is reported as June unless stated otherwise, month range reported in text. ^1^ Variable not included in the final regression model after model selection procedure.

**Table 2:** Seasonal effect on commercial tank milk stable isotope ratios (*n* = 217).

‰	Summer		Autumn		Winter		Spring		
	Mean	Range	Mean	Range	Mean	Range	Mean	Range	*P* value
δ^13^C	–26.6^a^	–30.1/ − 21.4	–26.2^ab^	–30.8/ − 21.4	–25.2^b^	–30.8/ − 20.7	–26.1^ab^	–30.8/ − 21.4	0.019
δ^15^N	5.52	3.92/ 7.78	5.80	4.36/ 7.45	5.63	4.43/ 8.04	5.83	4.43/ 8.20	0.186
δ^18^O	–5.80^b^	–8.94/ − 3.63	–7.05^a^	–10.8/ − 4.58	–8.43^c^	–12.8/ − 5.97	–6.87^a^	–11.5/ − 3.37	< 0.001
δ^2^H	–46.0^b^	–68.1/ − 32.0	–53.2^a^	–62.0/ − 42.1	–60.5^c^	–76.0/ − 46.9	–53.7^a^	–69.5/ − 35.7	< 0.001

P values = one way analysis of variance with season (categorical) as explanatory variable, ^a–d^ = means within rows with different superscripts are significantly different at *P* < 0.05.

### Associations between grassland-based feeds and stable isotope ratios in commercial tank milk

**Fig. 2:**
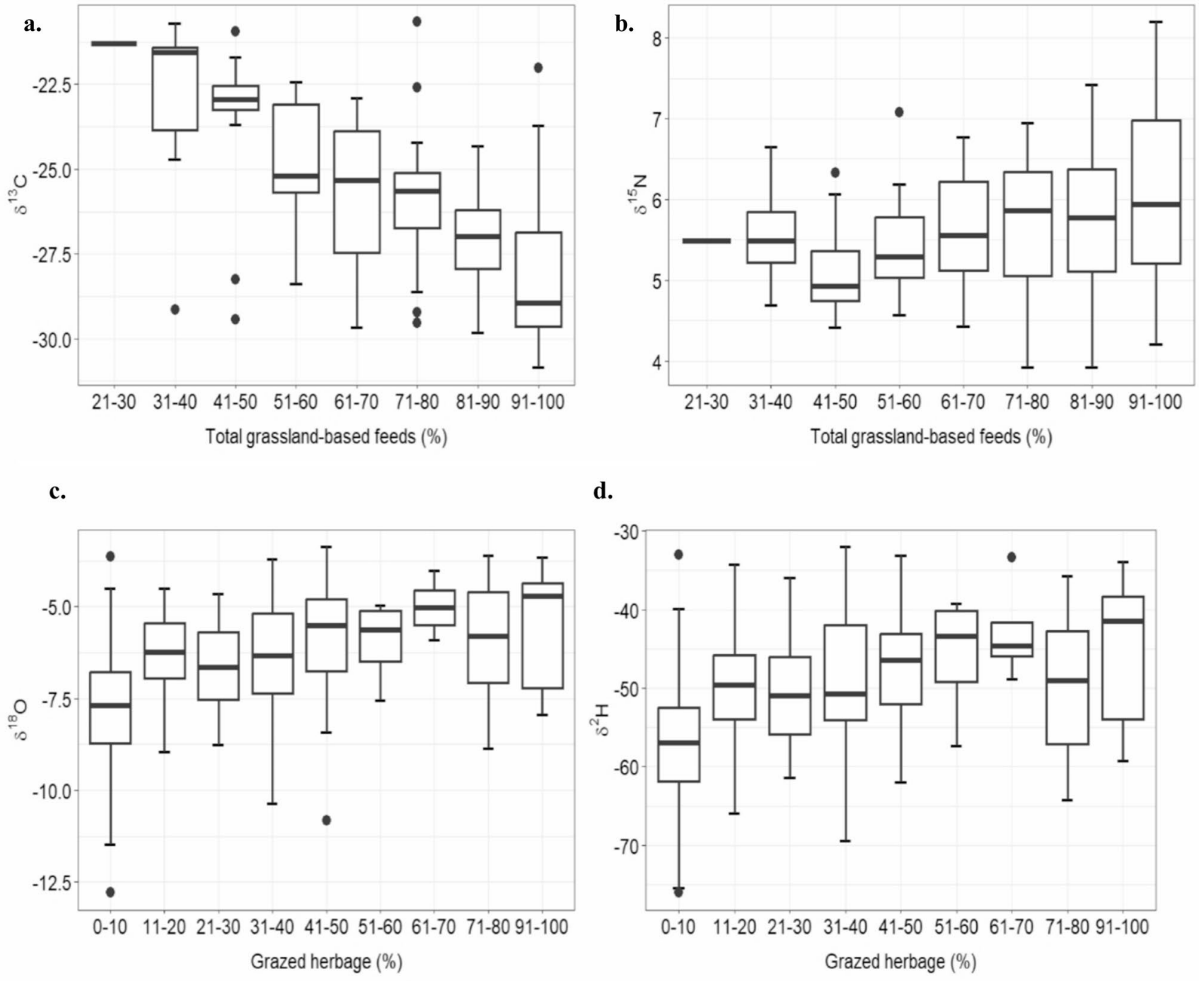
Effect of the proportion of total grassland-based feeds (C and N) as well as grazed herbage (O and H), in 10% increases, on (**a**) the δ^13^C values, (**b**) the δ^15^N values, (**c**) the δ^18^O values, and (**d**) the δ^2^H values of commercial tank milk.

**δ**^13^**C.** Milk δ^13^C values ranged from ‒30.9 to ‒20.7‰ across all farms, feeding strategies and seasons. Regression analysis revealed an associative effect (*P* < 0.001) of total GBF on commercial tank milk δ^13^C values (Table 1). The higher the proportion of GBF in the herd’s diet, the more negative the δ^13^C value of the milk became. Figure 2a illustrates the associative effect of the dietary proportion of total GBF, in 10% increases, on the δ^13^C value of the tank milk. In the model including grazed herbage, the months of May to September had a negative effect (*P* < 0.05) on milk δ^13^C values (Table 1); however, the fixed effects alone explained a relatively small proportion (marginal R^2^ = 0.10) of the total variance in δ^13^C. This suggests that the farm, included in the model as a random factor, was a significant factor in explaining the variance in milk δ^13^C (conditional R^2^ = 0.68) (Table 1). This may be attributed to the overall feeding strategy of the farms in terms of maize and concentrate feeds.

**δ**^15^**N**. Milk δ^15^N values ranged from 3.92 to 8.20‰ across all farms, feeding strategies and seasons. The proportion of total GBF in the herd’s diet did not have a significant associative effect on δ^15^N values (Table 1; Fig. 2b); however, there was a trend (*P* < 0.10) towards higher milk δ^15^N values with increasing proportions of grazed herbage in the diet. Milk δ^15^N values were higher (*P* < 0.01) in April compared to the other months of the year.

**δ**^18^**O.** Milk δ^18^O values ranged from ‒12.8 to ‒3.37‰ across all farms, feeding strategies and seasons. An increasing proportion of grazed herbage in the diet increased (*P* < 0.01) δ^18^O values in the tank milk (Table 1). This can be also seen in Fig. 2c where the associative effect of the proportion of grazed herbage, in 10% increases, is illustrated. However, milk δ^18^O values were not significantly affected by the proportion of total GBF in the diet. The months of April to October had a positive associative effect (*P* < 0.05) on milk δ^18^O values (Table 1). The location (Fig. 1) of the farm within Switzerland significantly affected milk δ^18^O values, where latitude had a positive effect (*P* < 0.05) on milk δ^18^O, while longitude had a negative effect (*P* < 0.05). The fixed effects alone explained a substantial portion of the total variance in milk δ^18^O values (marginal R^2^ = 0.45 and 0.47 for the two δ^18^O GBF models, total GBF and grazed herbage).

**δ**^2^**H.** Milk δ^2^H values ranged from ‒76.0 to ‒32.0‰ across all farms, feeding strategies and seasons. Milk δ^2^H values followed the same pattern. A higher proportion of grazed herbage in the herd’s diet had an increasing associative effect (*P* < 0.01) on the tank milk δ^2^H values (Table 1; Fig. 2d). The milk δ^2^H values were not significantly affected by the proportion of total GBF in the herd’s diet (Table 1). The months of April to October had a significant positive effect on milk δ^2^H values. Milk δ^2^H values were positively (*P* < 0.05) affected by latitude and negatively (*P* < 0.05) by longitude of the farm. The fixed effects alone explained a substantial portion of the total variance in milk δ^2^H values (marginal R^2^ = 0.48 and 0.50 for the two δ^2^H GBF models, total GBF and grazed herbage).

### Associations between maize (fresh and conserved) and stable isotope ratios in commercial tank milk

**Fig. 3:**
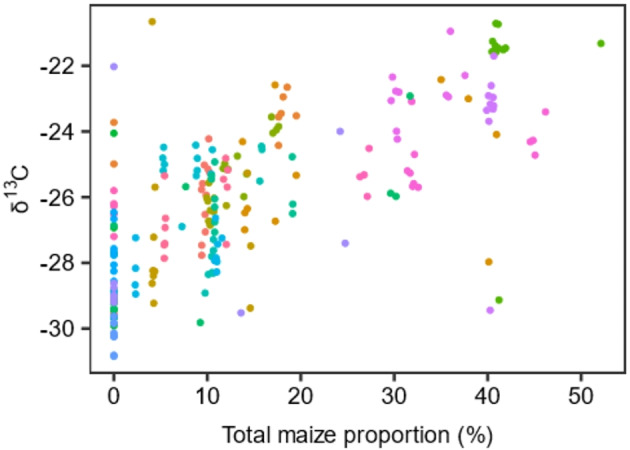
Effect of total maize proportion in % of total diet (each farm is represented by a different color, *n* = 21) on the individual δ^13^C values of commercial tank milk (*n* = 217).

Regression analysis revealed an associative effect, where an increasing proportion of maize in the dairy herd’s diet was associated with higher tank milk δ^13^C values. (*P* < 0.001; Table 1; Fig. 3). None of the other milk’s stable isotope ratios we tested (N, O, H) were significantly affected by the proportion of maize consumed by the herd. However, there was a trend (*P* < 0.10) towards increased milk δ^18^O values with increasing maize consumption at herd level (Table 1). Month was not included in the δ^13^C regression model based on maize proportion; however, in the δ^15^N model the month of April was most significant (*P* < 0.01) and in the δ^18^O and δ^2^H models, the months of April to September were most significant (*P* < 0.001). We found a negative interaction (*P* < 0.001) between month (July) and dietary maize proportion in the δ^18^O model. Regarding the location of the commercial dairy farms (Fig. 1), there was a trend (*P* < 0.10) for longitude to be positively correlated with the tank milk δ^15^N values while it was negatively correlated (*P* < 0.01) with the stable isotope ratios (O and H) in the models based on maize proportion (Table 1). Latitude was positively correlated (*P* < 0.05) with milk O and H stable isotope ratios.

### Seasonal effect on commercial tank milk stable isotopic profiles (C, N, O, H)

**Fig. 4:**
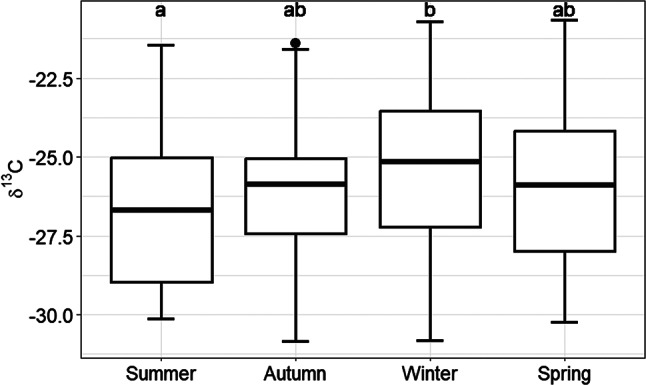
Seasonal effect on the δ^13^C values of commercial tank milk.

There was an effect (*P* < 0.05) of season for δ^13^C, δ^18^O and δ^2^H, but not for δ^15^N (Table 2). According to Tukey’s HSD post-hoc test (Table 2) the mean values for δ^13^C were lowest in summer (‒26.6 ± 2.42) and highest in winter (‒25.2 ± 2.29) (*P* < 0.05; Fig. 4). The mean milk water related isotope values (δ^18^O and δ^2^H) were higher (*P* < 0.05) in summer compared to autumn and spring and lower (*P* < 0.05) in winter compared to summer, autumn and spring (Table 2).

### Stable isotope ratio analysis as part of a future “sustainability indicator” for milk produced from grassland-based feeds

**Fig. 5:**
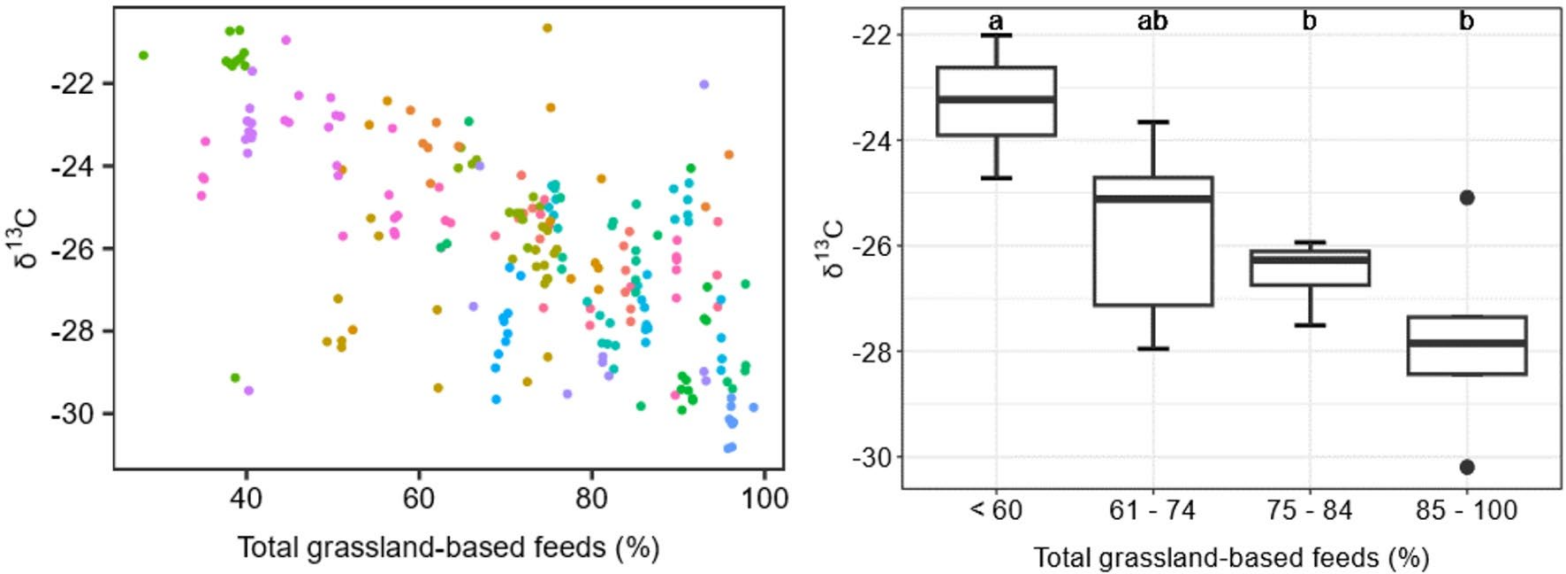
Effect of the farm feeding strategy (each farm is represented by a different color, *n* = 21), in terms of total proportion of grassland-based feeds, on the individual δ^13^C values of commercial tank milk (*n* = 217) (**a**) and the mean δ^13^C values of commercial tank milk compared with the mean proportion of total grassland-based feeds specified as one of four categories (**b**) (< 60% *n* = 4; 61–74% *n* = 5; 75–84% *n* = 7; 85–100% *n* = 5) for the year 2020.

Figure 5a depicts the associative effect total GBF (each farm is represented by a different color, *n* = 21) on the δ^13^C values of the tank milk (*n* = 217). There was also an associative effect (*P* = 0.001) of the grassland-based feeding category. In detail, the mean δ^13^C values of the tank milk on farms where the herds were fed a mean of < 60% GBF was different (*P* < 0.05) to the mean δ^13^C values where the herds received a mean of > 75% GBF in the diet (Fig. 5b). The other defined grassland-based feeding categories were not statistically different from each other.

## Discussion

### Associations between grassland-based feeds and stable isotope ratios in commercial tank milk

#### δ^13^C

 As the proportion of total GBF increased in dairy herd diets the δ^13^C values of the tank milk decreased. We found that for every 10% increase in GBF proportion, δ^13^C values decreased by 0.67‰ holding all other variables constant. While ours is the first study to consider binary proportions of GBF in the diet relative to δ^13^C values, Kornexl et al.^17^ and O’Sullivan et al.^16^ showed that milk from dairy herds fed GBF demonstrated lower δ^13^C than those fed maize and concentrates. Kornexl et al.^17^ recorded δ^13^C values in the range of − 26.5 to − 29.4‰ where the higher values were observed in winter according to the higher proportion of maize silage in the diet. O’Sullivan et al.^16^ reported mean values of − 25.8‰ for herds fed large amounts of concentrates (> 1,000 kg concentrates per head annually) and − 28.1‰ for those fed a primarily GBF diet (< 500 kg concentrates per head annually). This is because the photosynthetic pathways of C_3_ (temperate climate grasses) and C_4_ (maize and tropical grasses) plants fractionate C isotopes to different extents which is clearly evident in the δ^13^C values of C_3_ (~ − 20 to − 37‰) and C4 (~ − 10 to − 16‰) plants^18,26^. In the present study, the relationship between grazed herbage (0 to 100% across diets with a mean of 25.3%) and the δ^13^C values of the tank milk were not significant, this is probably because the grazed herbage proportion of total GBF was highly variable across farms and months. Nonetheless, the months of May to September had a significant and negative impact on δ^13^C values reflecting the average grazing periods in temperate climate countries.

#### δ^15^N

 While the δ^13^C value of commercial tank milk reflects the C_3_/C_4_ ratio of the herd’s diet, the δ^15^N values represent local conditions^17,27^. The δ^15^N values of the feed and subsequently the milk are mainly determined by the size and composition of the soil N pool (nitrate and ammonia) available to the growing plant^28^. In turn, the soil N pool is affected by the altitude of the area which affects the mean annual temperature and mean annual precipitation of the region^27^. The trend we found towards increasing δ^15^N values of the tank milk with increasing grazed herbage in the herd’s diet support those of O’Sullivan et al.^16^ who reported a mean increase of 7.13‰ in δ^15^N values in milk from primarily grass-fed cows compared to those fed high amounts of concentrates (> 1000 kg concentrate per head annually). Although longitude was significant in our model, it was not significant in the δ^15^N models presented by O’Sullivan et al.^16^. This leads us to believe that, as in the O’Sullivan et al.^16^ study, the fertilizer regime on the farms was the main cause of increasing δ^15^N values of the tank milk with increasing GBF. This assumption is strengthened as the month of April particularly promoted the δ^15^N values of the tank milk in our study, coinciding with the same month that grasslands are heavily fertilized in temperate climate countries and dairy herds are first turned out to pasture. Slurry, or organic fertilizer has a higher δ^15^N value (2 to 30‰) than mineral fertilizers (–4 to + 4‰)^11^. This is due to losses of volatile N compounds, mainly ammonia, as there is a strong fractionation. In Switzerland, slurry is the primary N source on grasslands as only about 20 to 40 kg N/ha/year from synthetic sources are typically applied^29,30^, hence the rather positive (3.9 to 8.3‰) δ^15^N values of the tank milk in our study.

#### Milk water related isotopes, δ^18^O and δ^2^H

 While the proportion of total GBF in the diet exhibited no clear relationship with the δ^18^O and δ^2^H values of the water fraction of the tank milk, the proportion of grazed herbage in the diet had a significant positive associative effect on the δ^18^O and δ^2^H values. With every 10% increase of grazed herbage in the diet the δ^18^O values of milk water increased by 0.125 holding all other variables constant. Our results support those of Garbaras et al.^32^ and O’Sullivan et al.^16^ who reported lower δ^18^O milk values in winter and higher δ^18^O milk values in summer. Grazing in Switzerland is restricted to the vegetation period from spring to autumn. Accordingly, milk water δ^18^O and δ^2^H values were significantly higher from April to October reflecting a seasonal and climatic effect. The most likely cause for this is the shift from primarily groundwater in the diet, via the tap water offered in the barn, to mostly plant water in the diet. Swiss groundwater has a fairly constant stable δ^18^O (–11.8 to − 9.5^31^), while in the warmer seasons, the additional source of water intake, grazed herbage, is enriched in^18^O^32^. Consistent with this, feeding dairy herds with fresh herbage enriched in^18^O due to evapotranspiration resulted in considerable increases in milk δ^18^O in the studies by Kornexl et al.^17^ and Bontempo et al.^33^.

### Associations between maize (fresh and conserved) and stable isotope ratios in commercial tank milk

As expected, our results revealed that the more whole plant maize (fresh and conserved) a dairy herd consumed, the higher the δ^13^C values of the tank milk became as C_3_ plants were exchanged with C_4_ plants in the diet. Sources of C_3_ plants in the dairy herd’s diet include not only GBF but also concentrates (assuming maize was not a major component) and other feed components such as wheat and barley. Therefore, relating the isotope ratios to dietary whole plant maize proportion carries additional information to that provided by GBF proportion. These results support those reported previously^16,17,19^ and mirror the findings by Camin et al.^19^ who demonstrated that for every 10% increase in maize in the herd’s diet, the δ^13^C values of milk casein increased by 0.7 to 1.0‰. We found similar values for whole milk: for every 10% increase in whole plant maize in the diet, the δ^13^C values of whole milk increased by 0.97‰. None of the other milk stable isotope ratios we tested (N, O, H) were significantly affected by the proportion of maize consumed by the herd; however, there was a trend towards higher milk δ^18^O values with increasing maize consumption at herd level particularly in the months of April to September. This is likely due to the^18^O enriched vegetal waters of the grazed herbage (see discussion above) and probably not a direct effect of the whole plant maize proportion in the diets. The interaction between dietary proportion of total GBF and the month of July reflected a negative effect on the δ^18^O values of the tank milk. Here a possible explanation may be the 17 days of rain in July 2020^34^. Precipitation water, depleted in^18^O, has a direct effect on cows’ drinking water and with increasing proportions of GBF consumed (in this case most likely grazed herbage), increasing amounts of water depleted in^18^O would have been concomitantly ingested ultimately affecting the δ^18^O and δ^2^H values of the tank milk.

### Seasonal effect of farm feeding strategy on commercial tank milk stable isotopic profiles

There was a clear effect of season on the δ^13^C, δ^18^O and δ^2^H values of commercial tank milk. Mean values of δ^13^C in the tank milk were lowest in summer and highest in winter. These results are in good agreement with those of Kornexl et al.^17^ and O’Sullivan et al.^16^ who found more positive δ^13^C in milk during the winter months. The most likely reason in all three studies is the increased maize and concentrate (which may include some maize) feeding over winter when fresh herbage supply is limited^16,17^. The mean milk water related isotope values (δ^18^O and δ^2^H) were significantly higher in the summer months and significantly lower in the winter months compared to the rest of the year. The values we report are in agreement with previous studies^16,17,32^ and are most likely due to the enrichment of vegetal waters with^18^O in the warmer months due to evapotranspiration^17,33^ and the change from mainly groundwater to water ingested through grazed herbage^32^ as discussed above.

### Stable isotope ratio analysis as part of a future “sustainability indicator” for milk produced from grassland-based feeds

As outlined in the introduction, grassland-based feeding is largely considered sustainable. Two decades ago, Pirog^35^ reported that about 10% of consumers were willing to pay ≥ 30% more for milk produced from “grass-fed” cows. Nowadays, a reported 64% of consumers are willing to pay more for grass fed dairy^36^ largely due to the animal welfare and human nutrition benefits attributed to this type of production. This raises the question, what is “grass-fed”? The verification of “grass-fed” in the diets of dairy cows is a global challenge as grass-based feeding regulations vary from country to country and label to label^20^. In many temperate-climate countries, year-round grazing is not possible as plummeting winter temperatures cannot sustain sufficient grass growth. However, conserved grass, such as silage and hay, hold much of the same “grass-fed” benefits associated with fresh grass. Therefore, we suggest that “grass-fed” should refer to the proportion of total GBF in the diets of dairy cows. This would allow for producers, consumers and policy makers to refer to a standard definition such as “75% grass-fed” etc. Camin et al.^19^ suggested a threshold value of − 23.5‰ for δ^13^C in milk casein, above which it is not possible to exclude the presence of maize in the diet. The δ^13^C value of whole milk is more negative than that of milk casein as it also contains the fat fraction which is more negative in general^17,18^. Therefore, primary results from the present study suggest that for whole milk this threshold may be set even lower at − 27.4‰ (y = − 27.4 + (9.71× Diet_c_) based on the whole milk δ^13^C regression to 0% whole plant maize in the diet (Table 1). However, this may be too low in practice as based on Fig. 3, farms feeding little to no maize by be unfairly discriminated against, a more realistic cut point may be around − 24‰ to account for noise in the data. Similar to the maize exclusion thresholds described above, GBF inclusion thresholds could conceivably be established. A such, a primary GBF inclusion threshold of − 26.0‰ (y = 40.6 + (– 6.71 × Diet_c_) + ( – 1.31 × average latitude in our study: 47.0) (based on the whole milk δ^13^C regression to 75% GBF in the diet, Table 1) may be suggested for δ^13^C, above which it is not possible to guarantee 75% GBF in the diet. This threshold, in combination with the maize threshold as a negative control, offers a possible first step towards the authentication of defined GBF proportions in dairy herd diets. In addition to setting and controlling thresholds, the δ^13^C value of commercial tank milk may be a suitable starting point for determining the proportion of GBF ingested by dairy herds due to the linear relationship observed between the δ^13^C value of the milk and the proportion of total GBF in the diet.

Dairy herds grazing on pasture, an element of total GBF ingestion, is considered an animal welfare marker and may particularly appeal to consumers as they demand more ethical production systems around animal products^37^. As such, the additional guarantee of a certain proportion of grazed herbage in the diet would be beneficial for milk producers and policy makers. Initial results revealed that as the proportion of grazed herbage in the diet increased, δ^18^O and δ^2^H became less depleted (i.e., lower proportions of the heavier^18^O and^2^H isotopes) in milk water. We found that for every 10% increase of grazed herbage in the diet the δ^18^O values of milk water are expected to increase by 0.125 holding all other variables constant. However, due to the variation in of δ^18^O values in the present study and considering this a first-step approach, more research is required to refine δ^18^O as a reliable grazed herbage indicator as in practice two samples differing by 0.1‰ cannot be distinguished.

### Defining the isotopic composition of commercial tank milk produced in Switzerland

#### δ^13^C 

The δ^13^C values of milk produced in Switzerland (− 30.9 to − 20.7‰, mean = − 26.0 ± 2.4) are generally lower than those of milk produced in the neighboring countries of France (− 21.9 ± 0.5)^38^, Germany (− 26.3 to − 22.0)^39^ and Italy (− 24.0 to − 17.2)^40^. This is likely due to the trend away from grazing and grass-based feeding systems across Europe as dairy production systems continue to intensify^41,42^. Accordingly, in countries dominated by intensive dairy production systems such as the US and China, the δ^13^C values of milk were far higher with − 21.2 (± 0.1) and − 16.0 (± 0.5), respectively^16^, than those of milk produced in Switzerland, likely reflecting the large amounts of maize and maize-based concentrates in the diet. However, the δ^13^C values of milk produced in Switzerland are very similar to the milk casein δ^13^C values of milk produced in Ireland (− 30.5 to − 20.3)^16^ and New Zealand (− 27.8 ± 0.7)^43^. This is not surprising as farmers in all three countries rely heavily on GBF for milk production^44–46^.

#### δ^15^N

 The δ^15^N values of milk produced in Switzerland (3.92 to 8.20‰, mean = 5.69 ± 0.86) are generally higher when compared to milk produced in the nearby region of Bavaria in Germany (3.5 to 5‰)^17^ and the neighboring countries of France (5.01 to 5.39‰) (reported as Europe in Dong et al.^47^) and Italy (4.4 to 5.8‰). This is probably due to the widespread use of slurry on grasslands^29,48^. However, as Switzerland experienced a particularly dry spell in April 2020^49^ these values could be over- or under-reported in our data as the lack of precipitation may have prevented slurry from adequately penetrating the soil resulting in lower-than-normal values. Alternatively, higher-than-normal values are conceivable if the lack of precipitation promoted slurry to adhere to grazed herbage.

#### Milk water related isotopes, δ^18^O and δ^2^H

 The δ^18^O (–12.8 to − 3.37‰, mean = − 7.03 ± 1.72) and δ^2^H (–76.0 to − 32.0‰, mean = − 53.4 ± 9.0) values of milk produced in Switzerland were very different to that of coastal countries such as Ireland (δ^18^O, − 0.85 to − 5.34‰)^16^ and a lot more similar to neighboring countries such as Northern Italy (δ^18^O, − 10.4 to − 5.23‰; δ^2^H, − 73.7 to − 39.0‰)^50^. This results from the loss of the heavier water isotopes through precipitation primarily near the coast, a phenomenon previously discussed by Franke et al.^51^ regarding the value of δ^18^O and δ^2^H for the geographic authentication of animal-sourced foods.

## Conclusions

Based on the results from the present study we suggest that δ^13^C and δ^18^O values of commercial tank milk may be suitable starting points for the development of reliable indicators to assess the proportion of total GBF and grazed herbage, respectively, in the diets of dairy herds. However, the reality is that although an isotopic approach for the determination of total GBF proportion in the diets of dairy herds may be accurate, it would be an added step for milk payment systems that commonly use mid-infrared spectroscopy to assess milk fat and protein contents, as well as a more expensive (± 8 USD per sample) approach that requires specific machinery and skilled personnel. However, as spot controls may be sufficient and only one sample per herd is required, the cost of this analysis could be acceptable. Additionally, our study provides initial data on the isotopic composition of milk produced in Switzerland which may allow differentiation from milk produced in other countries by the C, N, O and H stable isotope ratios of whole, raw milk and milk water. Limitations to this study include the relatively small sample size (*n* = 217), although great care was taken to ensure a representative selection of farms with varying feeding strategies. Larger international datasets are required to validate thresholds and progress research in this field.

## Electronic supplementary material

Below is the link to the electronic supplementary material.

Supplementary Material 1

## Data Availability

The datasets used and/or analyzed during the current study are available from the corresponding author on reasonable request.
