# Supplementary material for: Stable isotopic profile of commercial tank milk in relation to grassland based feed proportions in dairy herd diets
Source: Sci Rep. 2025 Apr 12;15:12694. doi: 10.1038/s41598-025-97041-z (PMC11993563; doi:10.1038/s41598-025-97041-z)
Supplement: Supplementary file 1 — Supplementary Material 1 [file 41598_2025_97041_MOESM1_ESM.pdf]

Supplementary Information

Stable isotopic profile (C, N, O, H) of commercial tank milk as affected by grassland-based feed proportions in dairy herd diets

Amy Birkinshaw,[1] Michael Sutter,[1] Rudi Schäufele[2], Michael Kreuzer[3] and Beat Reidy[1,✉]

[1]Bern University of Applied Sciences (BFH), School for Agricultural, Forest and Food Sciences (HAFL), Laenggasse 85, 3052 Zollikofen, Switzerland. [2]TUM School of Life Sciences, Crop Physiology, Alte Akademie 12, 85354 Freising, Germany. [3]ETH Zurich, Institute of Agricultural Sciences, Eschikon 27, 8315 Lindau, Switzerland. ✉Corresponding author: Beat Reidy, Bern University of Applied Sciences (BFH), School for Agricultural, Forest and Food Sciences (HAFL), Laenggasse 85, 3052 Zollikofen, Switzerland, Phone +41 31 910 22 23, e-mail: beat.reidy@bfh.ch

%	Total grassland-based feeds		Grazed herbage		Maize		Concentrates		Other feeds	
	Mean±SD	Range	Mean±SD	Range	Mean±SD	Range	Mean±SD	Range	Mean±SD	Range
Farm 1	78.8±6.10	71.5–84.5	14.0±13.5	0– 27.4	9.70±0.30	9.40–10.3	8.20±2.20	6.10–10.9	3.20±3.70	0– 7.30
Farm 2	69.7±15.4	59.0–95.9	30.4±42.3	0– 95.9	13.7±8.50	0– 19.5	13.2±5.20	4.10–17.6	3.50±2.20	0– 4.90
Farm 3	69.5±13.0	51.1–81.1	27.7±22.1	0– 47.2	24.0±11.7	13.8–40.9	6.50±1.40	5.10–8.70	0	0
Farm 4	59.8±10.1	49.3–74.9	33.3±29.8	0– 63.5	7.00±4.80	4.10–14.6	9.00±3.70	5.50–13.1	24.2±7.60	15.2–33.3
Farm 5	74.3±1.40	70.8–75.9	0	0	10.4±0.60	9.90–12.0	12.8±0.70	12.2–14.8	2.40±0.20	2.00–2.70
Farm 6	69.8±3.50	64.5–74.0	8.50±6.80	0– 14.9	14.4±2.40	11.7–17.6	15.9±1.90	13.5–18.5	0	0
Farm 7	37.8±3.30	28.1–39.8	0	0	42.1±3.40	40.5–52.1	14.1±0.40	13.7–14.7	6.10±0.10	6.00–6.20
Farm 8	91.6±1.10	90.4–93.4	60.3±43.1	0– 91.7	0	0	4.50±0.20	4.10–4.80	3.90±1.10	2.20–4.80
Farm 9	85.0±15.2	62.5–97.8	0	0	10.8±14.0	0– 31.7	4.20±1.90	2.20–7.30	0	0
Farm 10	82.0±3.90	76.4–85.1	33.3±23.0	0– 48.0	13.2±4.10	10.4–19.1	4.20±0.10	4.10–4.60	0.60±1.20	0– 3.00
Farm 11	79.8±2.90	75.7–82.7	20.9±15.4	0– 36.9	12.0±2.60	9.80–15.9	8.20±0.80	7.20–9.90	0	0
Farm 12	84.6±7.50	75.0–91.2	40.3±38.6	0– 77.4	7.70±2.10	5.20–10.5	7.70±9.40	0–19.6	0	0
Farm 13	89.4±4.40	85.8–95.0	0	0	7.90±4.40	2.30–11.6	2.70±0.00	2.70–2.80	0	0
Farm 14	69.9±0.90	68.8–71.8	5.70±3.90	0– 8.40	0	0	30.1±0.90	28.2–31.2	0	0
Farm 15	96.4±0.90	95.8– 8.7	53.6±37.4	0– 79.6	0	0	3.60±0.90	13.0–4.20	0	0
Farm 16	81.6±10.4	66.3–93.2	45.2±35.4	0– 79.5	7.00±10.9	0– 24.8	8.40±1.10	6.80–9.30	3.10±4.70	0– 9.40
Farm 17	40.3±0.30	39.9–40.7	0	0	40.3±0.30	39.9–40.7	19.3±0.50	18.7–20.2	0	0
Farm 18	48.2±2.80	44.4–50.9	7.80±6.70	0– 13.1	32.6±3.20	29.7–37.6	12.6±1.50	9.10–14.5	6.07±0.30	6.30–7.30
Farm 19	48.5±10.8	34.8–57.5	20.4±16.3	0– 33.8	36.8±6.70	31.4–46.2	13.0±2.30	10.8–16.3	1.70±2.40	0– 4.80
Farm 20	79.0±13.9	62.3–89.8	17.9±15.7	0– 34.2	10.8±13.9	0– 27.3	10.2±0.10	0– 10.4	0	0
Farm 21	81.3±9.40	68.8–94.6	23.5±23.1	0– 49.9	8.40±3.40	5.40–12.2	8.80±6.00	0– 15.6	1.50±1.70	0– 3.50

Supplementary Table S1. Overview of the main feed components per farm, n=21. SD, standard deviation.

‰	$\delta^{13}C$		$\delta^{15}N$		$\delta^{18}O$		$\delta^{2}H$	
	Mean ± SD	Range	Mean ± SD	Range	Mean ± SD	Range	Mean ± SD	Range
Farm 1	–26.0 ± 1.11	– 27.8 /– 24.2	5.94 ± 0.567	4.84 – 6.49	– 6.56 ± 0.81	– 7.49 /– 5.09	– 50.4 ± 3.87	– 54.2 /– 43.4
Farm 2	–23.7± 0.752	– 25.0 /– 22.7	5.94 ± 0.404	5.43 – 6.49	– 7.88 ± 1.04	– 9.87 /– 6.35	– 58.7 ± 4.82	– 68.5 /– 52.5
Farm 3	–25.1 ± 1.94	– 27.8 /– 22.4	4.98 ± 0.579	3.92 – 5.73	– 6.60 ± 1.407	– 8.88 /– 4.15	– 50.0 ± 8.83	– 60.3 /– 33.2
Farm 4	– 27.1 ± 2.52	– 29.4 /– 20.7	6.25 ± 0.474	5.70 – 7.08	– 6.92 ± 2.293	– 10.4 /– 4.62	– 53.9 ± 13.7	– 76.0 /– 39.3
Farm 5	– 26.2 ± 0.457	– 26.9 /– 25.5	6.50 ± 0.205	6.22 – 6.93	– 8.11 ± 0.991	– 10.1 /– 6.93	– 59.7 ± 3.47	– 66.6 /– 55.5
Farm 6	– 24.7 ± 0.753	– 26.0 /– 23.6	4.92 ± 0.452	3.92 – 5.58	– 6.75 ± 1.379	– 8.76/– 4.52	– 51.5 ± 9.52	– 64.4 /– 34.3
Farm 7	– 22.0 ± 2.38	– 29.1/– 20.7	5.76 ± 0.397	5.26 – 6.65	– 6.65 ± 0.848	– 8.35 /– 5.23	– 52.1 ± 3.00	– 56.4 /– 48.9
Farm 8	– 28.4 ± 1.75	– 29.9 /– 24.1	6.24 ± 0.975	4.63 – 8.20	– 6.19 ± 2.079	– 9.22 /– 3.66	– 49.0 ± 11.4	– 65.8 /– 34.0
Farm 9	– 27.4 ± 2.245	– 29.8 /– 22.9	4.68 ± 0.376	4.21 – 5.50	– 6.89 ± 2.117	– 10.3 /– 3.63	– 50.6 ± 9.3	– 63.8 /– 33.1
Farm 10	– 25.9 ± 0.778	– 27.1 /– 24.8	5.68 ± 0.584	5.02 – 6.45	– 7.03 ± 2.94	– 12.8 /– 3.37	– 51.9 ± 10.8	– 75.5 /– 40.0
Farm 11	– 27.1 ± 1.65	– 28.9 /– 24.5	6.21 ± 0.371	5.77 – 7.09	– 6.13 ± 1.90	– 8.73 /– 3.71	– 48.2 ± 9.34	– 61.8 /– 36.0
Farm 12	– 25.1 ± 0.683	– 26.9 /– 24.4	5.69 ± 0.481	4.87 – 6.40	– 7.75 ± 0.86	– 8.87 /– 6.09	– 59.1 ± 4.15	– 64.2 /– 50.2
Farm 13	– 27.9 ± 0.677	– 28.9 /– 26.6	6.58 ± 0.679	5.21 – 7.42	– 7.63 ± 1.69	– 9.79 /– 4.64	– 57.1 ± 9.41	– 70.0 /– 41.2
Farm 14	– 28.0 ± 0.967	– 29.7 /– 26.5	6.10 ± 0.642	4.58 – 6.77	– 8.05 ± 1.58	– 10.9 /– 5.46	– 59.8 ± 9.00	– 71.5 /– 40.0
Farm 15	– 30.2 ± 0.417	– 30.8 /– 29.6	7.63 ± 0.356	6.98 – 8.18	– 5.84 ± 1.75	– 8.71 /– 3.83	– 49.7 ± 8.15	– 61.0 /– 37.4
Farm 16	– 27.5 ± 2.66	– 29.5 /– 22.0	5.67 ± 0.501	4.55 – 6.26	– 6.79 ± 2.79	– 11.5 /– 3.62	– 50.6 ± 13.7	– 67.5 /– 33.3
Farm 17	– 23.6 ± 2.11	– 29.4 /– 21.7	5.11 ± 0.566	4.42 – 6.33	– 6.90 ± 0.94	– 8.94 /– 5.89	– 51.7 ± 4.67	– 63.7 /– 47.8
Farm 18	– 22.8 ± 0.910	– 24.2 /– 21.0	4.84 ± 0.319	4.47 – 5.36	– 7.00 ± 1.25	– 9.03 /– 5.45	– 51.6 ± 4.57	– 57.1 /– 44.7
Farm 19	– 24.7 ± 0.889	– 25.7 /– 23.1	5.17 ± 0.255	4.68 – 5.51	– 7.58 ± 1.67	– 10.4 /– 4.82	– 56.6 ± 8.81	– 69.5 /– 39.0
Farm 20	– 26.3 ± 1.37	– 29.6 /– 24.5	4.89 ± 0.478	4.22 – 5.73	– 7.06 ± 1.81	– 9.53 /– 3.76	– 53.8 ± 11.6	– 65.4 /– 32.0
Farm 21	– 26.4 ± 1.10	– 27.9 /– 24.8	4.97 ± 0.306	4.43 – 5.52	– 7.30 ± 1.35	– 10.3 /– 5.52	– 54.5 ± 6.40	– 66.1/ – 44.3

Supplementary Table S2. Detailed isotopic profile of bovine milk for each farm, n=21. SD, standard deviation.
